# Supplementary material for: The postcranial skeleton of Cerrejonisuchus improcerus (Crocodyliformes: Dyrosauridae) and the unusual anatomy of dyrosaurids
Source: PeerJ. 2021 May 10;9:e11222. doi: 10.7717/peerj.11222 (PMC8117932; doi:10.7717/peerj.11222)
Supplement: Supplemental Information 1 [file peerj-09-11222-s001.pdf]

SUPPLEMENTARY INFORMATION

A MEASUREMENTS

We used laser (Creaform Handyscan 300) and white light (Artec Eva) surface scanners to acquire additional measurements on 3D models, using the software GOM Inspect 2019. We laser scanned all the elements of the holotype of *Congosaurus bequaerti* (MRAC 1741–1743, 1745, 1796, 1797, 1799, 1802, 1803, 1806, 1809–11, 1813–1819, 1822–1832 (A, B & C), 1835 (A, B & C)-1841, 1844–1846, 1848–1858, 1860–1874, 1876, 1877, 1879, 1882–1884, 1887–1896), at resolution ranging from 0.3 to 0.5 mm, depending on the size of the element. We surface scanned *Hyposaurus natator* (NJSM 23368) using structured white light with error down to 0.5 mm, and this scan was complemented by digital caliper measurements of the cast replica of both femora and humeri and from measurements gathered prior to the mounting of the specimen (W. Callahan pers. comm. 14 August 2019).

B CROCODYLIFORM HABITATS

The habitat of the fossil crocodyliforms analyzed ('thanatocoenosis' on our graphs) were either directly taken or inferred from literature. Here is a list of our species and the corresponding literature for their habitat:

- *Anthracosuchus balrogus* in Hastings et al. [2014]
- *Cerrejonisuchus improcerus* in Hastings et al. [2010, 2011]
- *Dyrosaurus maghribensis* in Jouve et al. [2006] (plus Yans et al. [2014])
- *Hyposaurus natator* (*Hyposaurus rogersii* is now a *nomen dubium* see Jouve et al. [2020]) from Denton et al. [1997], Wilberg et al. [2019]
- *Terminonaris browni* [Osborn, 1904] in Wu et al. [2001], Adams et al. [2011], Wilberg et al. [2019]
- *Congosaurus bequaerti* Schwarz et al. [2006], Schwarz-Wings et al. [2009], Wilberg et al. [2019]
- *Thoracosaurus neocesariensis* (De Kay, 1842) in Carpenter [1983], Wilberg et al. [2019]
- *Gavialosuchus/Thecachampsa americana* in Lara [2012]
- *Metriorhynchus moreli* in Buffetaut [1981], Young et al. [2010], Wilberg et al. [2019]
- *Suchodus durobrivensis* in Buffetaut [1981], Young et al. [2010], Wilberg et al. [2019]
- *Lemmysuchus obtusidens* in Buffetaut [1981], Wilberg et al. [2019]
- *Steneosaurus bollensis* in Buffetaut [1981], Wilberg et al. [2019]
- *Metriorhynchus cultridens* in Buffetaut [1981], Young et al. [2010], Wilberg et al. [2019]
- *Suchodus superciliosus* in Buffetaut [1981], Young et al. [2010], Wilberg et al. [2019]

Cerrejonisuchus improcerus is interpreted as 'less aquatic than *Acherontisuchus guajiraensis*' according to Hastings et al. [2011], so we placed it under the 'terrestrial' label but only to oppose it to the aquatic-freshwater lifestyle inferred for *Acherontisuchus guajiraensis*. *Cerrejonisuchus improcerus* is not understood as fully terrestrial, but is here more viewed as *Osteolaemus tetraspis*.

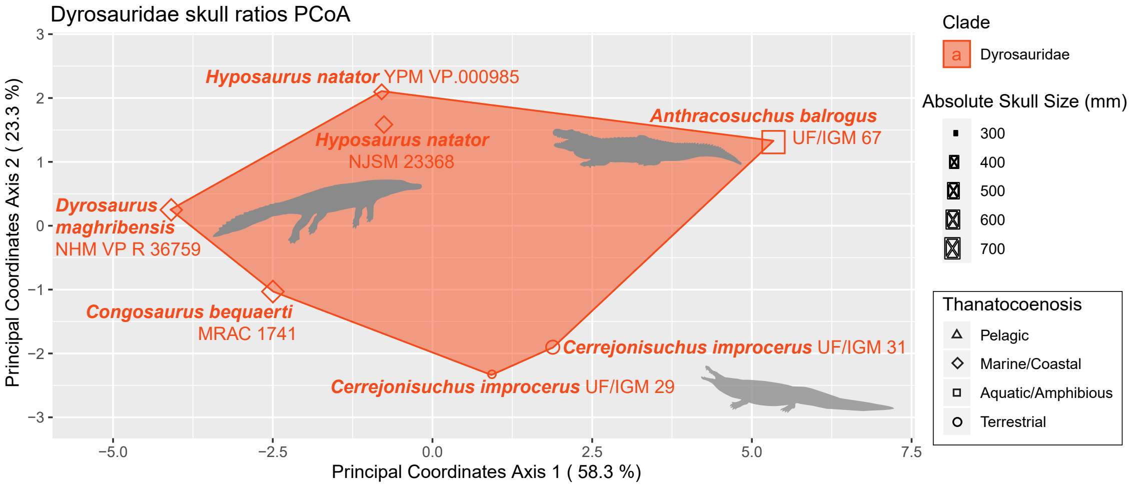

Figure SI 1. Morphospace representing dissimilarity between dyrosaurid taxa using the first two PCoA axes, based on the dyrosaurid skull ratio dataset. Polygon encompasses Dyrosauridae members while colored symbols illustrate lifestyles. The relative size of the lifestyle symbols depends on the absolute skull size of the specimen. *Hyposaurus vector* by Nobu Tamura (vectorized by Zimices); *Anthracosuchus* vector by Roberto D ’iaz Sibaja

C DYROSAURID PCOA

In order to assess more specifically the morphological variations among dyrosaurids, two additional datasets were composed and analyzed: the first one only comprised skull ratios (25 ratios - columns, of which 5 are area ratios), and the second one only had postcranial ratios (170 ratios - columns, of which 73 are area ratios). Statistical analyses were conducted following the exact same method as described in the main article.

As the osteological and taxonomic status of the NJSM 12293 specimen are unclear [Morgan et al., 2018, Denton et al., 1997], we have decided to only stipulate its inventory number in our analyses.

The skull-dataset comprises 11 dyrosaurid specimens (11 rows) and 25 skull ratios (25 columns, of which 5 are area ratios), and has a total of 55.27% of missing data initially. This number is reduced to 27.47% after application of a completeness threshold of 25% for the characters and 50% for the taxa. The list of individuals of this dataset is available below, see table SI 2. Completeness thresholds of 25% and 50% have been respectively applied to columns and rows to obtain a complete distance matrix (*i.e.* 0% of missing values). The results are presented on fig. SI 1.

The postcranial-dataset comprises 40 dyrosaurid specimens (40 rows) and 170 postcranial ratios (170 columns, of which 73 are area ratios), and has a total of 91.25% of missing data initially. This number is reduced to 35.76% after application of a completeness threshold of 10% for the characters and 40% for the taxa. The list of individuals of this dataset is available below, see table SI 3. Completeness thresholds of 10% and 40% have been respectively applied to columns and rows to obtain a complete distance matrix (*i.e.* 0% of missing values). The results are presented on fig. SI 2.

On fig. SI 1, the dyrosaurids seem to be distributed as a function of their phylogenetic relationships (see Hastings et al. [2014], Wilberg et al. [2019], or it could be strongly influenced by longirostry): it is possible to draw a line on the first axis to separate the basal (right of graph) from the derived dyrosaurids (left of graph). Furthermore, *Anthracosuchus balrogus* and *Cerrejonisuchus improcerus* are found on two distinct poles, which means that they can be told apart from the second axis as well.

Yet, the major difference with the dyrosaurid phylogeny is the absence of a hyposaurine cluster: here *Congosaurus bequaerti* is more dissimilar to *Hyposaurus natator* than it is to *Dyrosaurus maghribensis*.

Hyposaurine dyrosaurids are grouped together on the left portion of the graph (fig. SI 2), with the

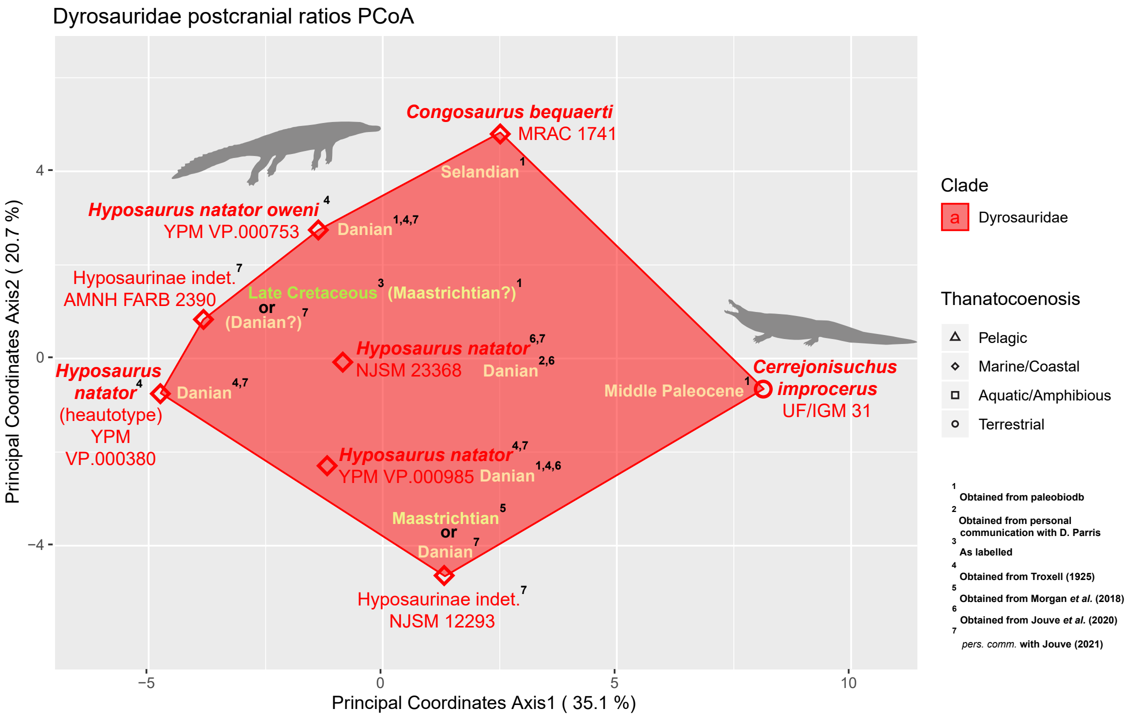

Figure SI 2. Morphospace representing dissimilarity between dyrosaurid taxa using the first two PCoA axes, with the dyrosaurid body ratio dataset. Polygon encompasses Dyrosauridae members while colored symbols illustrate lifestyles. *Hyposaurus vector* by Nobu Tamura (vectorized by Zimices).

Danian [Jouve et al., 2020] specimens of *Hyposaurus natator* (NJSM 23368 and YPM VP.000985 respectively) occupying center positions of the convex hull. *Cerrejonisuchus improcerus* is isolated and appears mostly dissimilar to *Hyposaurus* spp. The specimens that occupy the closest position to *Cerrejonisuchus improcerus* are the NJSM 12293 specimen and the holotype of *Congosaurus bequaerti*. As a result, the pattern of morphospace occupation reflect a phylogenetical clustering along the first axis, but could also reflect an environmental lifestyle splitting.

The *Hyposaurus* species are spaced so that the holotype specimen (YPM VP.000985) and the heautotype specimen (YPM VP.000380) of *Hyposaurus natator* are close to one another, while the specimen YPM VP.000753 appears more distant to them. This specimen was previously attributed to distinct subspecies, *Hyposaurus natator oweni* [Troxell, 1925]. A thorough investigation of the postcranial material previously (see Jouve et al. [2020]) referred to as *Hyposaurus* appears now necessary [Jouve et al., 2020]. It is possible that it might reveal species-level differences within this assemblage.

D THE SKULL

The skull of UF/IGM 31 (see fig. SI 14 in Supplementary Information), while not totally complete, strongly resembles that of the holotype UF/IGM 29 which is figured and extensively described in Hastings et al. [2010]. Here the dorsal skull length of UF/IGM 31 reaches 386 mm from the tip of the snout (premaxillae) to the quadrate, making it a longer specimen than the holotype UF/IGM 29 [Hastings et al., 2010], which is 299.5 mm long.

The whole dorsal surface of the skull bears shallow pits. The snout is short (179 mm from tip of the snout to the orbits), and reaches approximately 46% of the total skull length. There are two major snout constrictions: the first one near the anterior margin of the nasal cavity, the second one directly posterior to the premaxillary. These constrictions give room for large mandibular teeth. The premaxillary has four alveoli, whereas the maxillary possesses eleven alveoli. The orbits are anterodorsally oriented, and their shape is oval with the major axis mediolaterally oriented They are bordered by the lacrimal and prefrontal anteriorly, by the jugal lateral, by the frontal medially, and by the postorbital posteriorly. The supratemporal fenestra are reduced (about 59% of the infratemporal fenestra length), and close to the orbits. The supra temporal fenestra is limited by the postorbital anteriolaterally, by the frontal and parietal medially, and the squamosal posterolaterally. The nasal cavity is positioned anteriorly (near the skull margin), and is oval with the minor axis anteroposteriorly oriented. The nasal cavity is enclosed within the premaxillary (and thus does not meet with the nasal bone). The infratemporal fenestra starts at about the mid length of the supratemporal fenestra, and is mainly formed by the squamosal, jugal and quadratojugal (there is a presumed contribution of the postorbital).

E ADDITIONAL FIGURES

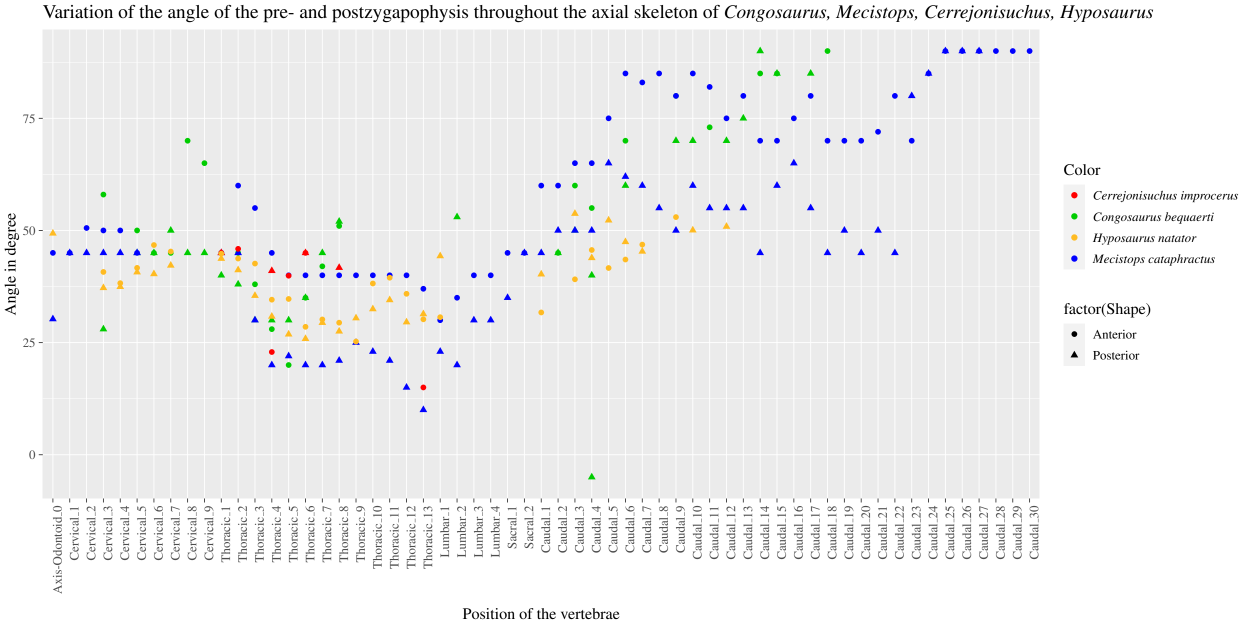

Figure SI 3. Evolution of the inclination angle in degree ° of the pre- and postzygapophysis throughout the axial skeletons of: *Congosaurus bequaerti*, *Mecistops cataphractus* (RBNIS 18374), *Hyposaurus natator* (NJSM 23368), and *Cerrejonisuchus improcerus* (UF/IGM 31). The angle is taken from the coronal plane. Position of each vertebrae is listed on the abscissa axis.

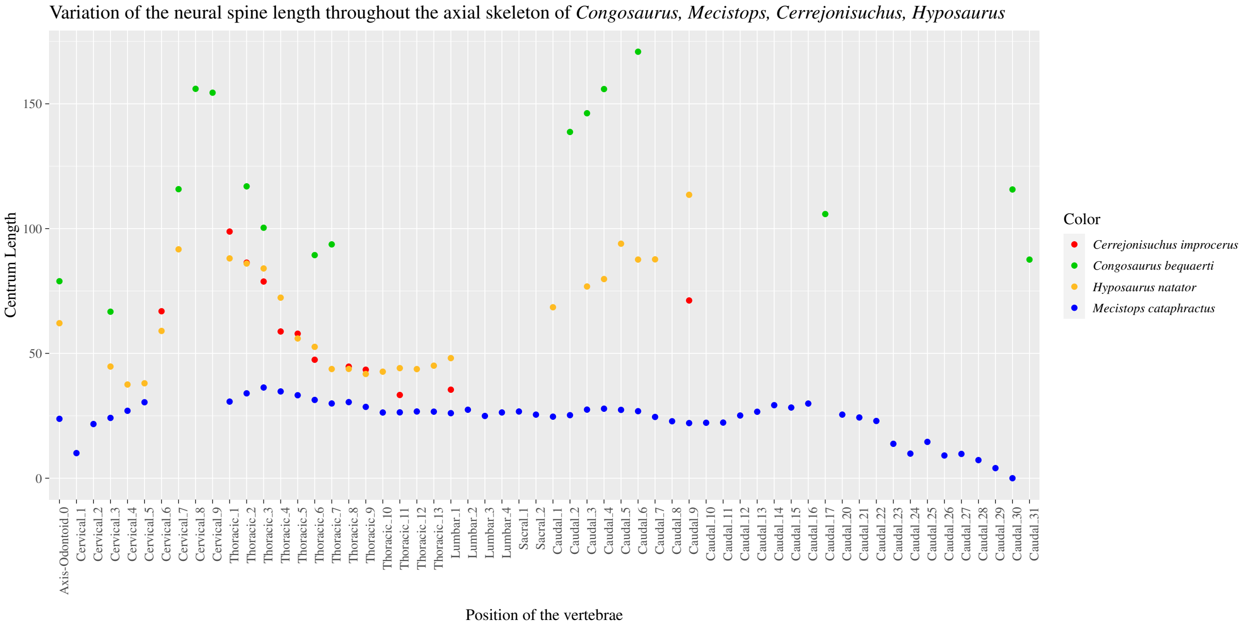

Figure SI 4. Scatter plot of the neural height variation throughout the axial skeleton of *Congosaurus bequaerti*, *Mecistops cataphractus* (RBNIS 18374), *Hyposaurus natator* (NJSM 23368), and *Cerrejonisuchus improcerus* (UF/IGM 31). Position of each vertebrae listed on the abscissa axis.

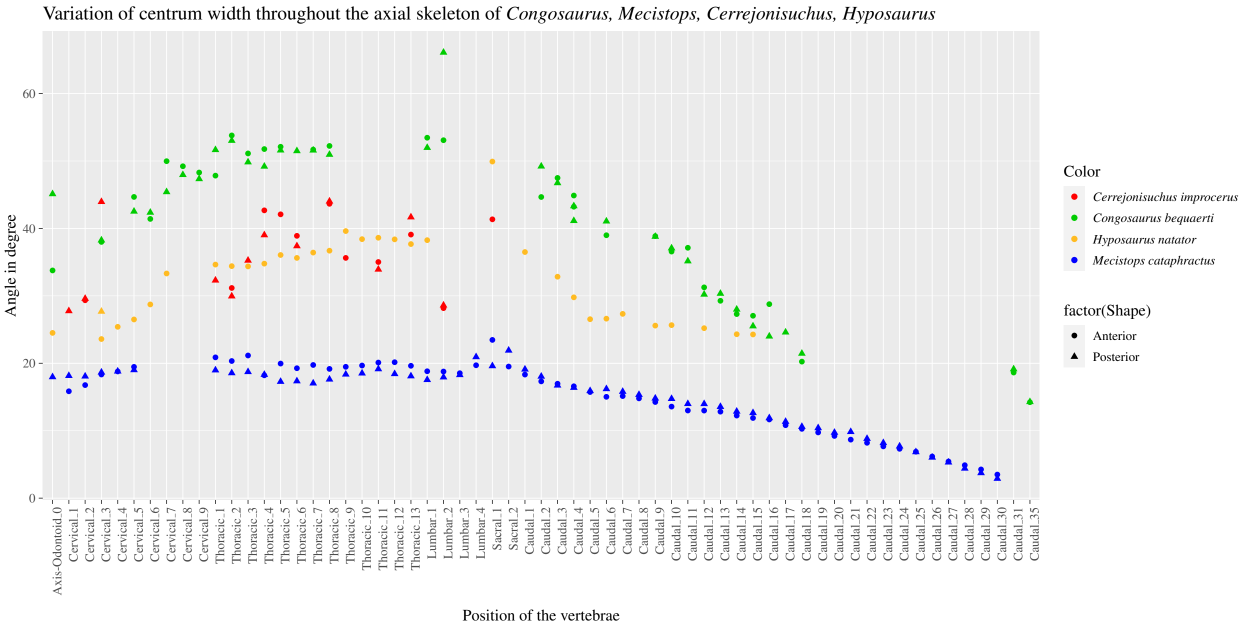

Figure SI 5. Scatter plot of the centrum width variation throughout the axial skeleton of *Congosaurus bequaerti*, *Mecistops cataphractus* (RBNIS 18374), *Hyposaurus natator* (NJSM 23368), and *Cerrejonisuchus improcerus* (UF/IGM 31). Position of each vertebrae listed on the abscissa axis.

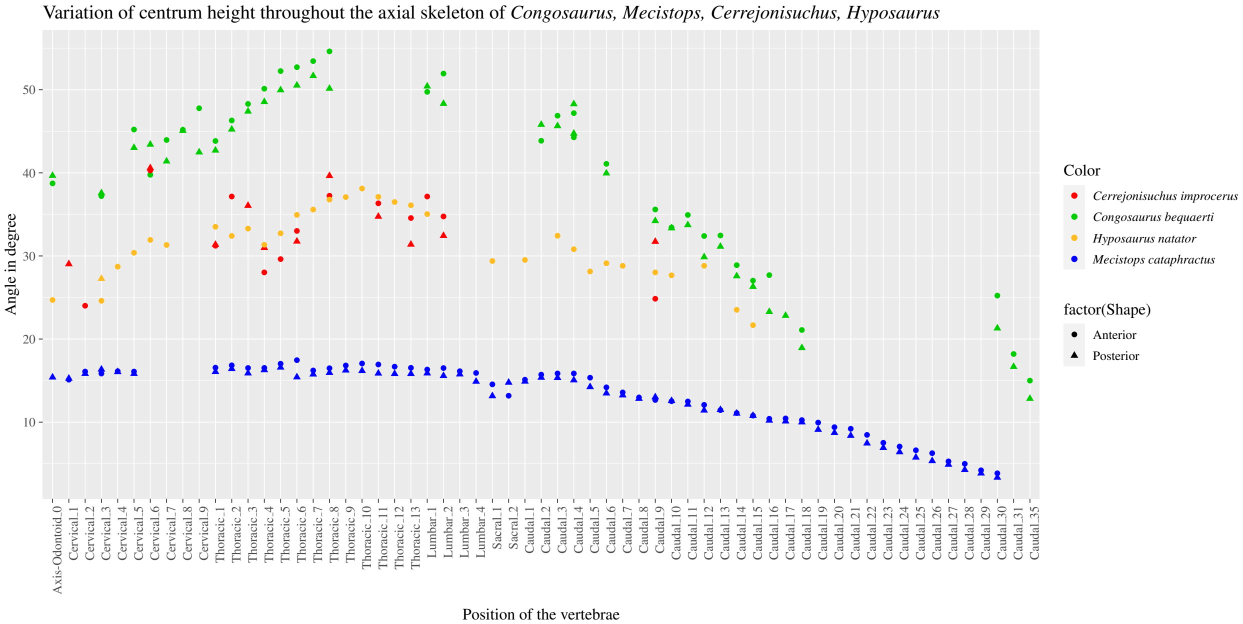

Figure SI 6. Scatter plot of the centrum height variation throughout the axial skeleton of *Congosaurus bequaerti*, *Mecistops cataphractus* (RBNIS 18374), *Hyposaurus natator* (NJSM 23368), and *Cerrejonisuchus improcerus* (UF/IGM 31). Position of each vertebrae listed on the abscissa axis.

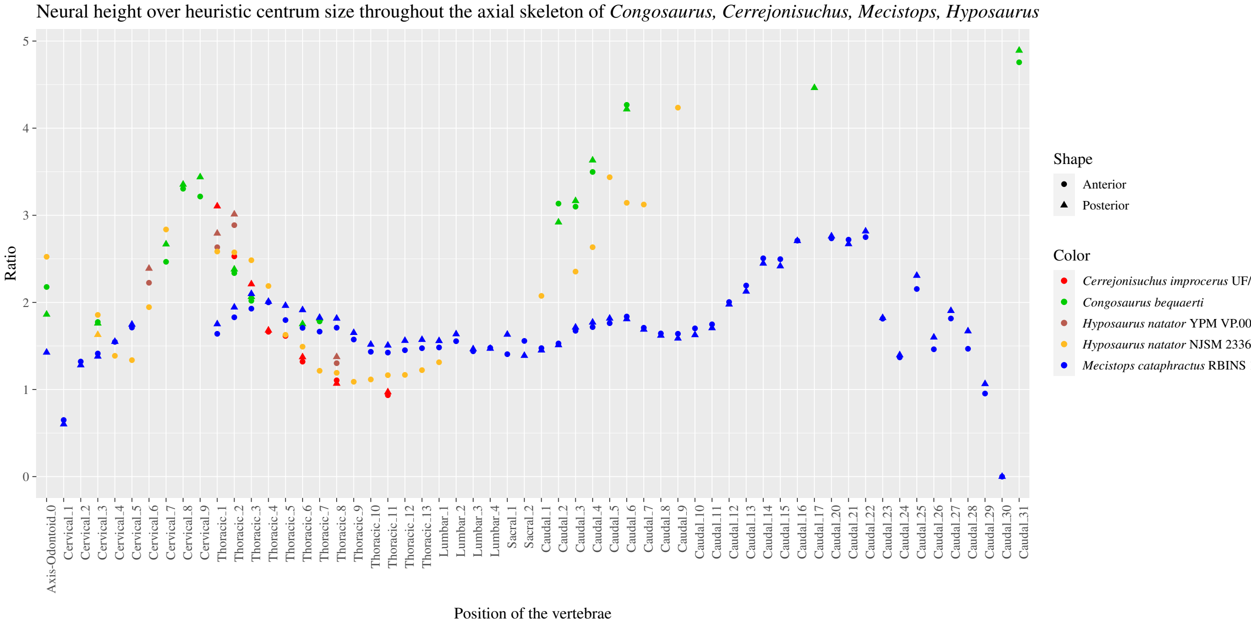

Figure SI 7. Estimation via heuristic of the neural spine height variation throughout the axial skeleton of *Congosaurus bequaerti*, *Hyposaurus natator* (NJSM 23368 and YPM VP.000985), *Cerrejonisuchus improcerus* (UF/IGM 31), and *Mecistops cataphractus* (RBNIS 18374). Position of each vertebrae listed on the abscissa axis.

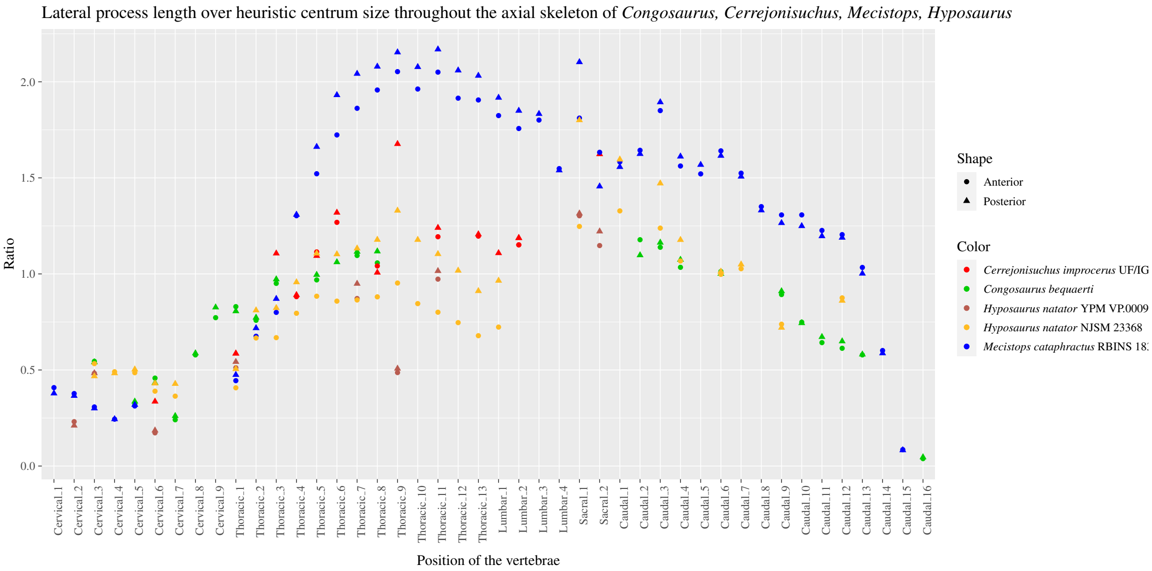

Figure SI 8. Estimation via heuristic of the maximal lateral process length variation throughout the axial skeleton of *Congosaurus bequaerti*, *Hyposaurus natator* (NJSM 23368 and YPM VP.000985), *Cerrejonisuchus improcerus* (UF/IGM 31), and *Mecistops cataphractus* (RBNIS 18374). Position of each vertebrae listed on the abscissa axis.

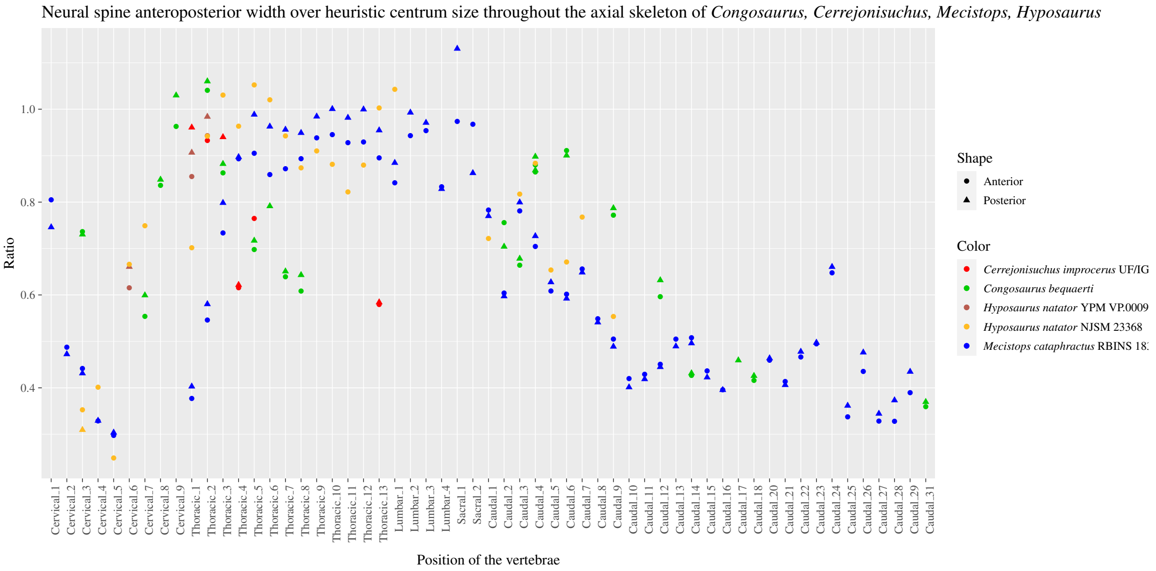

Figure SI 9. Estimation via heuristic of the neural spine anteroposterior width variation throughout the axial skeleton of *Congosaurus bequaerti*, *Hyposaurus natator* (NJSM 23368 and YPM VP.000985), *Cerrejonisuchus improcerus* (UF/IGM 31), and *Mecistops cataphractus* (RBNIS 18374). Position of each vertebrae listed on the abscissa axis.

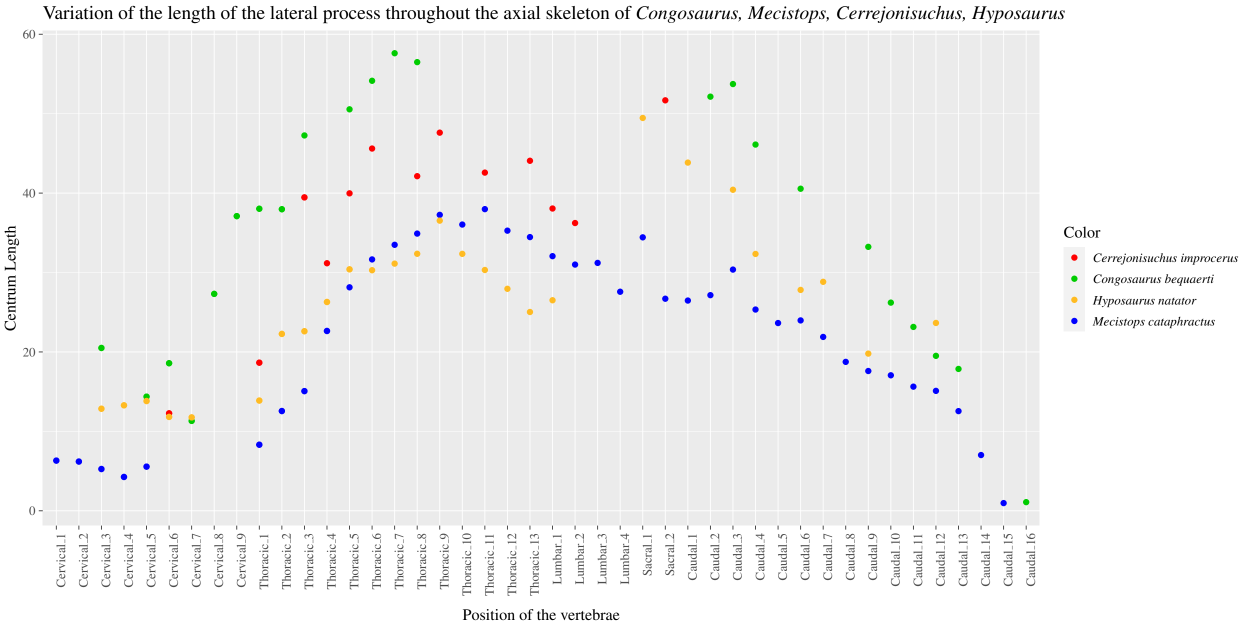

Figure SI 10. Scatter plot of the variation of the lateral process maximal length throughout the axial skeleton of *Congosaurus bequaerti*, *Mecistops cataphractus* (RBNIS 18374), *Hyposaurus natator* (NSJM 23368), and *Cerrejonisuchus improcerus* (UF/IGM 31). Position of each vertebrae listed on the abscissa axis.

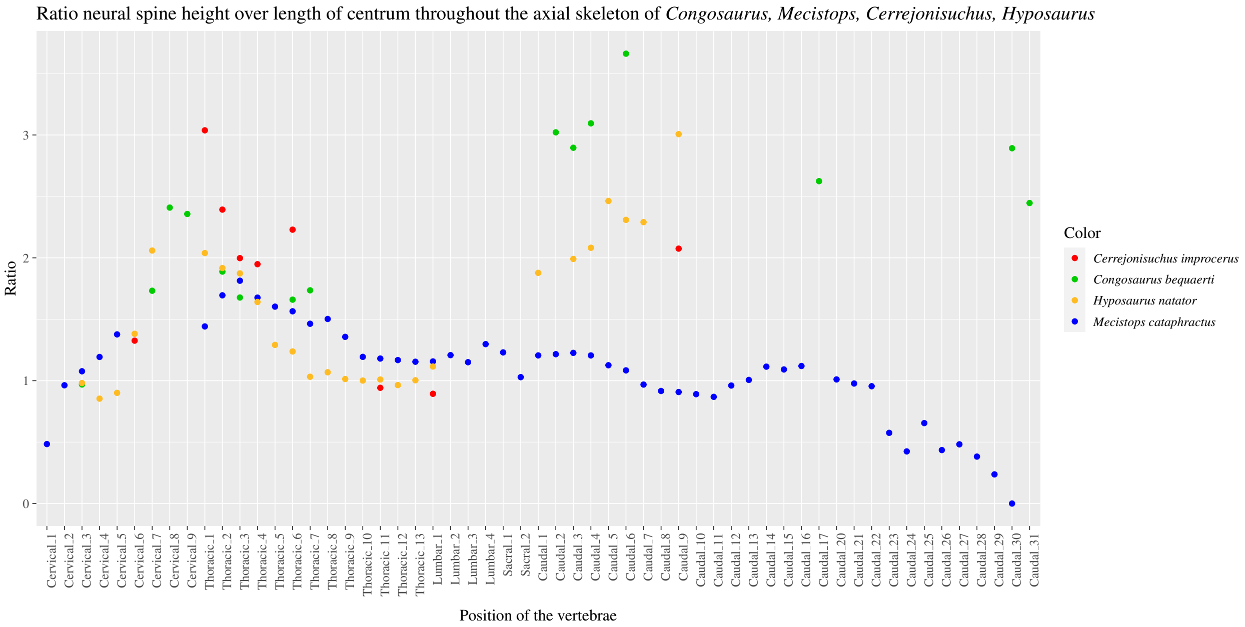

Figure SI 11. Scatter plot of the variation of ratio of the neural spine height over the centrum length throughout the axial skeleton of *Congosaurus bequaerti, Mecistops cataphractus* (RBNIS 18374), *Hyposaurus natator* (NSJM 23368), and *Cerrejonisuchus improcerus* (UF/IGM 31). Position of each vertebrae listed on the abscissa axis.

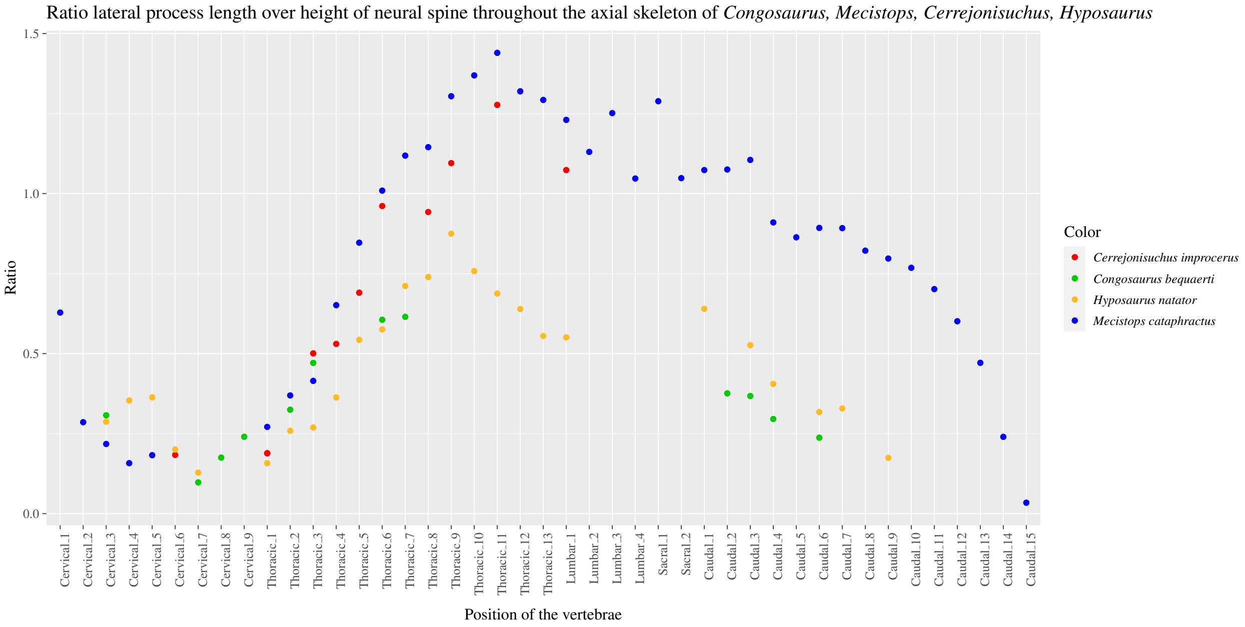

Figure SI 12. Scatter plot of the variation of ratio of the lateral process length over the neural spine height throughout the axial skeleton of *Congosaurus bequaerti, Mecistops cataphractus* (RBNIS 18374), *Hyposaurus natator* (NSJM 23368), and *Cerrejonisuchus improcerus* (UF/IGM 31). Position of each vertebrae listed on the abscissa axis.

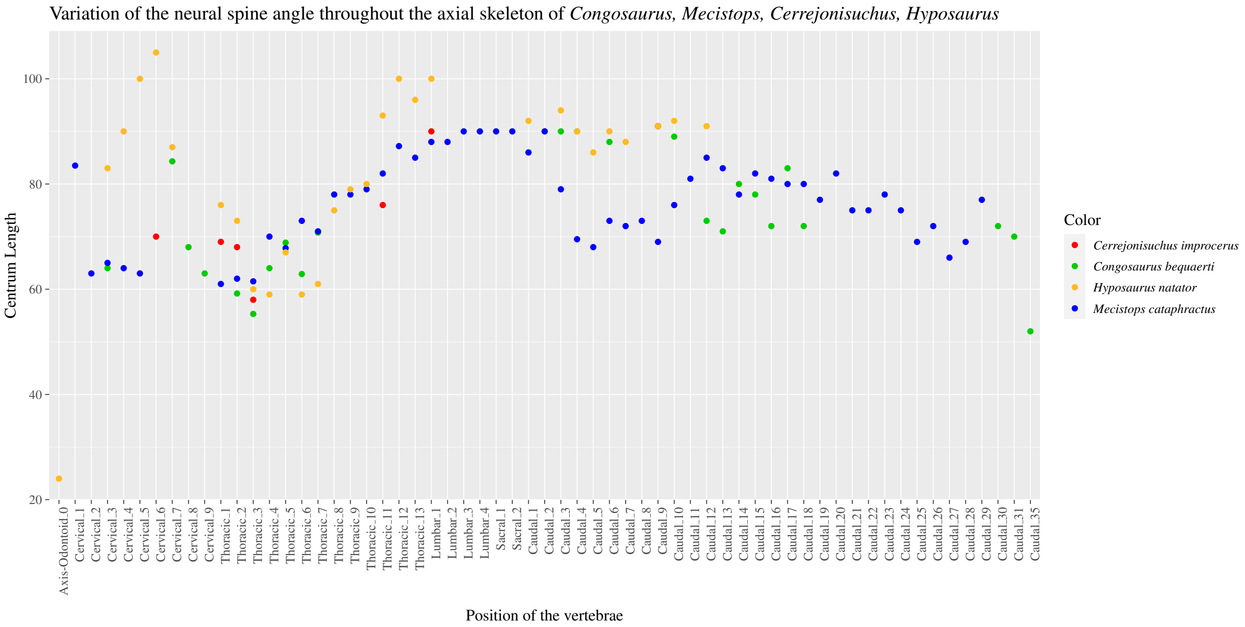

Figure SI 13. Scatter plot of the variation of the angle (in degrees) of the neural spine to the coronal plane throughout the axial skeleton of *Congosaurus bequaerti, Mecistops cataphractus* (RBNIS 18374), *Hyposaurus natator* (NSJM 23368), and *Cerrejonisuchus improcerus* (UF/IGM 31). Position of each vertebrae listed on the abscissa axis.

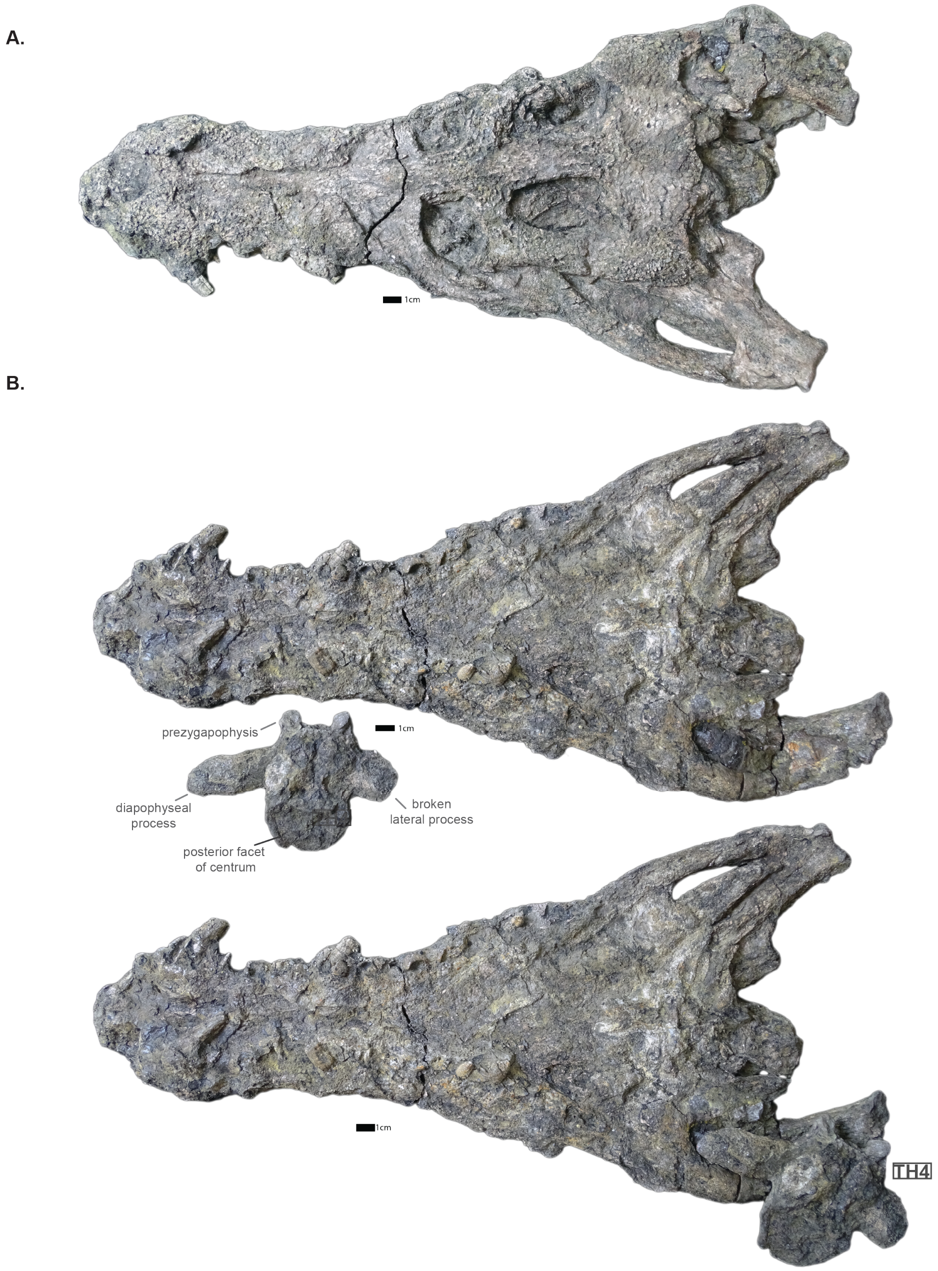

Figure SI 14. Skull and anterior thoracic vertebrae of *Cerrejonisuchus improcerus* UF/IGM 31. Thoracic vertebrae is in posterior view. **A.** dorsal view of the skull; **B.** ventral view of the skull.

F LISTS OF INDIVIDUALS

	Species	Inventory number
1	*Cerrejonisuchus improcerus*	UF/IGM 31
2	*Congosaurus bequaerti*	MRAC 1741
3	*Hyposaurus natator*	NJSM 23368
4	Hyposaurinae indet.	YPM VP.000764
5	*Anthracosuchus balrogus*	UF/IGM 68
6	*Anthracosuchus balrogus*	UF/IGM 67
7	*Metriorhynchus cultridens*	NHM VP R3804
8	*Lemmysuchus obtusidens*	NHM VP R3168
9	*Crocodylus rhombifer*	AMNH FARB 16624
10	*Hyposaurus natator*	YPM VP.000985
11	*Hyposaurus natator*	YPM VP.000380
12	*Hyposaurus natator oweni*	YPM VP.000753
13	*Mecistops cataphractus*	RBINS 18374
14	*Platysuchus multiscrobiculatus*	SMNS9930
15	*Steneosaurus bollensis*	SMNS 9428
16	*Pelagosaurus typus*	SMNS 80065
17	*Metriorhynchus moreli*	SMNS 10116
18	*Machimosaurus hugii*	SMNS 81608
19	*Dacosaurus maximus*	SMNS 8203
20	*Machimosaurus buffetauti*	SMNS 91415
21	*Crocodylus porosus*	R.G. 294
22	Cf. *Gavialosuchus americanus* or *Thecachampsa americana* ˜Brochu	ANSP 9369
23	*Terminonaris browni*	AMNH FARB 5844
24	*Suchodus superciliosus*	AMNH FARB 997
25	*Thoracosaurus neocesariensis*	NJSM 15437
26	*Dyrosaurus maghribensis*	NHM VP R 36759
27	*Suchodus durobrivensis*	NHM VP R 2618

Table SI 1. Total list of crocodyliform specimens (27) before application of completeness thresholds from the main analysis.

	Species	Inventory number
1	*Congosaurus bequaerti*	MRAC 1741
2	*Cerrejonisuchus improcerus*	UF/IGM 29
3	*Cerrejonisuchus improcerus*	UF/IGM 31
4	*Cerrejonisuchus improcerus*	UF/IGM 32
5	*Anthracosuchus balrogus*	UF/IGM 67
6	*Anthracosuchus balrogus*	UF/IGM 69
7	*Hyposaurus rogersii*	ANSP 8631;8629
8	*Hyposaurus natator*	YPM VP.000985
9	*Hyposaurus rogersii*	NJSM 10861
10	*Dyrosaurus maghribensis*	NHM VP R 36759
11	*Hyposaurus rogersii*	NJSM 23368

Table SI 2. Total list of dyrosaurid individuals before application of completeness thresholds in the cranial analysis

	Species	Inventory number
1	*Congosaurus bequaerti*	MRAC 1741
2	*Cerrejonisuchus improcerus*	UF/IGM 29
3	*Cerrejonisuchus improcerus*	UF/IGM 31
4	*Cerrejonisuchus improcerus*	UF/IGM 32
5	*Cerrejonisuchus improcerus*	UF/IGM 30
6	*Anthracosuchus balrogus*	UF/IGM 67
7	*Acherontisuchus guajiraensis*	UF/IGM 35
8	*Acherontisuchus guajiraensis*	UF/IGM 38
9	*Acherontisuchus guajiraensis*	UF/IGM 34
10	*Anthracosuchus balrogus*	UF/IGM 69
11	*Acherontisuchus guajiraensis*	UF/IGM 37
12	Hyposaurinae indet.	ANSP 8651
13	Hyposaurinae indet.	ANSP 8638
14	Hyposaurinae indet.	ANSP 8648
15	Hyposaurinae indet.	ANSP 8635
16	Hyposaurinae indet.	ANSP 8631;8629
17	Hyposaurinae indet.	ANSP 9682-93
18	Hyposaurinae indet.	ANSP 9631-79
19	Hyposaurinae indet.	ANSP 15364
20	Hyposaurinae indet.	ANSP 13656
21	Hyposaurinae indet.	AMNH FARB 1421
22	Hyposaurinae indet.	AMNH FARB 1416
23	Hyposaurinae indet.	AMNH FARB 1432
24	Hyposaurinae indet.	AMNH FARB 1432 19205
25	Hyposaurinae indet.	AMNH FARB 1432
26	Hyposaurinae indet.	AMNH FARB 2389-2390
27	Hyposaurinae indet.	AMNH FARB 2390
28	Hyposaurinae indet.	AMNH FARB 2390
29	Hyposaurinae indet.	AMNH FARB 19205
30	Hyposaurinae indet.	AMNH FARB 2202
31	Hyposaurinae indet.	AMNH FARB 19204
32	Hyposaurinae indet.	AMNH FARB 1459
33	*Hyposaurus natator*	YPM VP.000985
34	*Hyposaurus natator*	YPM VP.000380
35	*Hyposaurus natator (oweni)*	YPM VP.000753
36	*Hyposaurus* sp.	YPM VP.000764
37	*Hyposaurus natator*	NJSM 23368
38	Hyposaurinae indet.	NJSM 12293
39	*Hyposaurus* sp.	NJSM 10861
40	*Dyrosaurus maghribensis*	NHM VP R 36759

Table SI 3. Total list of dyrosaurid specimens (40) before application of completeness thresholds in the postcranial analysis.

105

Package	Use
APE	Multivariate analysis, PcoA
VEGAN	Test the statistical significance of the clusters w/ PERMANOVA (Permutational Multivariate Analysis of Variance Using Distance Matrices)
PSYCH	Functions for multivariate analysis
DENDEXTEND	Cut a Tree (Dendrogram/hclust/phylo) into Groups of Data from the cluster dendrogram results (clust_result); perform tanglegram
PVCLUST	Creates a tree to represent the ecomorpho groups that exist in our matrix data.s
DBI	To communicate with sqlite database
GGPLOT 2	To plot figures
GGNEWSCALE	To have more than one colour or fill scale in a plot using ggplot2

Table SI 4. Table depicting R packages employed.

REFERENCES

T. L. Adams, M. J. Polcyn, O. Mateus, D. A. Winkler, and L. L. Jacobs. First occurrence of the long-snouted crocodyliform *Terminonaris* (Pholidosauridae) from the Woodbine Formation (Cenomanian) of Texas. *Journal of Vertebrate Paleontology*, 31(3):712–716, 2011. ISSN 02724634. doi: 10.1080/02724634.2011.572938.

E. Buffetaut. Radiation évolutive, paléoécologie et biogéographie des crocodiliens mésosuchiens. *Mémoire de la Société Géologique de France*, 142:88, 1981.

K. Carpenter. *Thoracosaurus neocesariensis* (De Kay, 1842) (Crocodylia: Crocodylidae) from the Late Cretaceous Ripley Formation of Mississippi. *Mississippi Geology*, 4:10, 1983. ISSN 1098-6596. doi: 10.1017/CBO9781107415324.004.

R. K. Denton, J. L. Dobie, and D. C. Parris. The marine crocodilian *Hyposaurus* in North America. *Ancient Marine Reptiles*, pages 375–397, 1997. doi: 10.1016/b978-012155210-7/50020-x.

A. K. Hastings, J. I. Bloch, E. A. Cadena, and C. A. Jaramillo. A new small short-snouted dyrosaurid (Crocodylomorpha, Mesoeucrocodylia) from the Paleocene of Northeastern Colombia. *Journal of Vertebrate Paleontology*, 30(1):139–162, 2010.

A. K. Hastings, J. I. Bloch, and C. A. Jaramillo. A new longirostrine dyrosaurid (Crocodylomorpha, Mesoeucrocodylia) from the Paleocene of north-eastern Colombia: biogeographic and behavioural implications for New-World Dyrosauridae. *Palaeontology*, 54(5):1095–1116, 2011. ISSN 00310239. doi: 10.1111/j.1475-4983.2011.01092.x.

A. K. Hastings, J. I. Bloch, and C. Jaramillo. A new blunt-snouted dyrosaurid, *Anthracosuchus balrogus* gen. et sp. nov. (Crocodylomorpha, Mesoeucrocodylia), from the Palaeocene of Colombia. *Historical Biology*, pages 1–23, 2014.

S. Jouve, M. Iarochène, B. Bouya, and M. Amaghzaz. A new species of *Dyrosaurus* (Crocodylomorpha, Dyrosauridae) from the early Eocene of Morocco: phylogenetic implications. *Zoological Journal of the Linnean Society*, 148:603–656, 2006.

S. Jouve, C. de Muizon, R. Cespedes-Paz, V. Sossa-Soruco, and S. Knoll. The longirostrine crocodyliforms from Bolivia and their evolution through the Cretaceous–Palaeogene boundary. *Zoological Journal of the Linnean Society*, pages 1–35, 2020. ISSN 0024-4082. doi: 10.1093/zoolinnean/zlaa081.

J. V. T. Lara. Florida vertebrate fossils *Thecachampsa americana*, 2012. URL `https://www.floridamuseum.ufl.edu/florida-vertebrate-fossils/species/thecachampsa-americana/`.

D. J. Morgan, R. K. Denton, and R. E. Weems. Presence of a dyrosaurid neosuchian in the Severn/Brightseat Formation of Maryland. *The Mosasaur The Journal of the Delaware Valley Paleontological Society*, 10:91–104, 2018.

H. F. Osborn. Teleorhinus browni, a teleosaur in the Fort Benton. *Bulletin of the American Museum of Natural History*, 20:239–240, 1904.

D. Schwarz, E. Frey, and T. Martin. The postcranial skeleton of the Hyposaurinae (Dyrosauridae; Crocodyliformes). *Palaeontology*, 49(4):695–718, 2006.

D. Schwarz-Wings, E. Frey, and T. Martin. Reconstruction of the Bracing System of the Trunk and Tail in Hyposaurine Dyrosaurids (Crocodylomorpha; Mesoeucrocodylia). *Journal of Vertebrate Paleontology*, 29(2):453–472, 2009.

E. L. Troxell. *Hyposaurus*, a marine crocodilian. *American Journal of Science*, 9(54):489–514, 1925.

E. W. Wilberg, A. H. Turner, and C. A. Brochu. Evolutionary structure and timing of major habitat shifts in Crocodylomorpha. *Scientific reports*, 9(514):1–10, 2019. doi: 10.1038/s41598-018-36795-1.

X.-C. Wu, A. P. Russell, and S. L. Cumbaa. *Terminonaris* (Archosauria: Crocodyliformes): new material from Saskatchewan, Canada, and comments on its phylogenetic relationships. *Journal of Vertebrate Paleontology*, 21(3):492–514, 2001. ISSN 0272-4634. doi: 10.1671/0272-4634(2001)021.

J. Yans, M. Amaghzaz, B. Bouya, H. Cappetta, P. Iacumin, L. Kocsis, M. Mouflih, O. Selloum, S. Sen, J. Y. Storme, and E. Gheerbrant. First carbon isotope chemostratigraphy of the Ouled Abdoun phosphate Basin, Morocco; implications for dating and evolution of earliest African placental mammals. *Gondwana Research*, 25(1):257–269, 2014. ISSN 1342937X. doi: 10.1016/j.gr.2013.04.004.

M. T. Young, S. L. Brusatte, M. Ruta, and M. B. de Andrade. The evolution of Metriorhynchoidea (Mesoeucrocodylia, Thalattosuchia): an integrated approach using geometric morphometrics, analysis of disparity, and biomechanics. *Zoological Journal of the Linnean Society2*, 158:801–859, 2010.
